# Ubiquity, diversity and physiological characteristics of *Geodermatophilaceae* in Shapotou National Desert Ecological Reserve

**DOI:** 10.3389/fmicb.2015.01059

**Published:** 2015-09-30

**Authors:** Hong-Min Sun, Tao Zhang, Li-Yan Yu, Keya Sen, Yu-Qin Zhang

**Affiliations:** ^1^Institute of Medicinal Biotechnology, Chinese Academy of Medical Sciences and Peking Union Medical CollegeBeijing, China; ^2^Division of Biological Sciences, School of Science, Technology, Engineering, and Mathematics, University of Washington BothellBothell, WA, USA

**Keywords:** *Geodermatophilaceae*, 16S rRNA, diversity, physiological characteristics, desert

## Abstract

The goal of this study was to gain insight into the diversity of culturable actinobacteria in desert soil crusts and to determine the physiological characteristics of the predominant actinobacterial group in these crusts. Culture-dependent method was employed to obtain actinobacterial strains from desert soil samples collected from Shapotou National Desert Ecological Reserve (NDER) located in Tengger Desert, China. A total of 376 actinobacterial strains were isolated and 16S rRNA gene sequences analysis indicated that these isolates belonged to 29 genera within 18 families, among which the members of the family *Geodermatophilaceae* were predominant. The combination of 16S rRNA gene information and the phenotypic data allowed these newly-isolated *Geodermatophilaceae* members to be classified into 33 “species clusters,” 11 of which represented hitherto unrecognized species. Fermentation broths from 19.7% of the isolated strains showed activity in at least one of the six screens for antibiotic activity. These isolates exhibited bio-diversity in enzymatic characteristics and carbon utilization profiles. The physiological characteristics of the isolates from different types of crusts or bare sand samples were specific to their respective micro-ecological environments. Our study revealed that members of the family *Geodermatophilaceae* were ubiquitous, abundant, and diverse in Shapotou NDER, and these strains may represent a new major group of potential functional actinobacteria in desert soil.

## Introduction

It has become increasingly clear that the overuse of antibiotics and the subsequent rise in antibiotic-resistant pathogens will force us to search for new antibiotics to meet urgent clinical needs (Talbot et al., 2006). Previous studies have indicated that environments considered to be extreme habitats are rich sources of novel actinobacteria (Subramani and Aalbersberg, 2013). It has been hypothesized that unusual climate conditions and ecological factors may endow the organisms in such habitats with the unique capacity to produce novel bioactive compounds (Bull et al., 2005; Okoro et al., 2008).

The Shapotou desert region (latitude 36°39′-37°41′N, elevation 104°25′-105°40′E) is recognized as the first “National Desert Ecological Reserve” (NDER) in China. This NDER is renowned worldwide as a teaching and scientific research base for studying controlled desertification. It is located on the southeast edge of the Tengger Desert, south of the Yellow River, in the northwest part of China. This region is at an altitude of 1300–1700 m, has an annual average precipitation of 186.2 mm, an annual mean temperature of 9.7°C, and an annual average wind speed of 2.8 m/s with a typical temperate desert climate.

In desert regions, microbiotic crusts play a significant role in controlling desertification by providing surface stability. Microbiotic crusts are important in stabilization of the sandy surface, soil formation, and in carbon and nitrogen assimilation (Evans and Johansen, 1999). Microbiotic crusts in Shapotou NDER are generally categorized into the following three typical types: Cyanobacteria-dominated crusts (CC), Moss-dominated crusts (MC), and Lichen-dominated crusts (LC). Samples were therefore, collected from these three types of crusts and bare sands. Culture-dependent method was employed to evaluate the diversity of culturable actinobacteria in Shapotou NDER, and to explore the potential functional actinobacterial resources from this extreme environment.

Actinobacterial strains were discovered and identified from the three types of soil crusts and bare sands samples from the Shapotou NDER. We found that the members of the family *Geodermatophilaceae* were ubiquitous in the different types of crusts, as well as the bare sands samples. Based on the physiological characteristics of these diverse *Geodermatophilaceae* members, we characterized the influence of micro-ecological niche environments on the phenotypic characteristics of these isolates.

## Materials and methods

### Sample collection

A total of 50 samples for isolation of actinobacteria were collected from four different micro-ecological environments in Shapotou NDER (latitude 36°39′-37°41′N, elevation 104°25′-105°40′E). The detailed information regarding the sample number, type of sample, and specific collection location of the 50 samples is displayed in Table 1. All the samples were placed in sterilized envelopes following collection and taken to the laboratory within 1 week of collection. All samples were immediately processed for isolation after arriving at the laboratory.

**Table 1:** **Samples collected in the Shapotou region**.

**Sample number**	**Sample type**	**Site information**	**Sample number**	**Sample type**	**Site information**	**Sample number**	**Sample type**	**Site information**
BCL12001	BS	37°25′38.72″N	MSY12018	MS	37°27′38.50″N	BSY12035	BS	37°27′38.66″N
		104°35′8.13″E			104°59′58.79″E			104°59′58.52″E
		1701 mH			1329 mH			1329 mH
MCL12002	MS	37°25′37.85″N	MSY12019	MS	37°27′38.36″N	CSY12036	CC	37°27′38.12″N
		104°35′8.26″E			104°59′59.10″E			104°59′58.79″E
		1701 mH			1329 mH			1329 mH
BCL12003	BS	37°25′38.89″N	MSY12020	MS	37°27′38.18″N	CSY12037	CC	37°27′37.81″N
		104°35′9.01″E			104°59′59.54″E			104°59′59.47″E
		1701 mH			1329 mH			1329 mH
CCL12004	CC	37°25′38.65″N	MSY12021	MS	37°27′38.04″N	CSY12038	CC	37°27′37.76″N
		104°35′7.67″E			104°59′59.84″E			104°59′59.72″E
		1701 mH			1329 mH			1329 mH
MCL12005	MS	37°25′39.09″N	MSY12022	MS	37°27′37.86″N	CSY12039	CC	37°27′37.60″N
		104°35′7.97″E			105°00′0.17″E			104°59′59.72″E
		1701 mH			1329 mH			1329 mH
CYW12006	CC	37°25′30.86″N	MSY12023	MS	37°27′37.89″N	CSY12040	CC	37°27′37.40″N
		104°43′52.00″E			105°00′0.51″E			104°59′59.86″E
		1698 mH			1329 mH			1329 mH
LYW12007	LC	37°25′30.76″N	MSY12024	MS	37°27′38.09″N	CSY12041	CC	37°27′37.45″N
		104°43′53.52″E			105°00′0.28″E			105°00′0.13″E
		1698 mH			1329 mH			1329 mH
LYW12008	LC	37°25′29.83″N	MSY12025	MS	37°27′38.29″N	BSY12042	BS	37°27′37.29″N
		104°43′53.65″E			105°00′0.04″E			104°59′59.86″E
		1698 mH			1329 mH			1329 mH
BYW12009	BS	37°25′30.14″N	BSY12026	BS	37°27′38.63″N	BSY12043	BS	37°27′37.32″N
		104°43′51.19″E			104°59′59.68″E			104°59′59.59″E
		1698 mH			1329 mH			1329 mH
MYW12010	MS	37°25′31.17″N	CSY12027	CC	37°27′38.87″N	CSY12044	CC	37°27′37.34″N
		104°43′51.13″E			104°59′59.44″E			104°59′59.18″E
		1698 mH			1329 mH			1329 mH
BYW12011	BS	37°25′31.35″N	MSY12028	MS	37°27′39.09″N	BSY12045	BS	37°27′37.36″N
		104°43′52.09″E			104°59′59.24″E			104°59′58.79″E
		1698 mH			1329 mH			1329 mH
BHW12012	BS	37°27′3.06″N	MSY12029	MS	37°27′39.16″N	CSY12046	CC	37°27′37.55″N
		104°47′41.19″E			104°59′59.63″E			104°59′58.97″E
		1619 mH			1329 mH			1329 mH
BHW12013	BS	37°27′3.45″N	CSY12030	CC	37°27′39.03″N	CSY12047	CC	37°27′37.76″N
		104°47′42.21″E			104°59′59.89″E			104°59′58.65″E
		1619 mH			1329 mH			1329 mH
MHW12014	MS	37°27′3.67″N	MSY12031	MS	37°27′38.83″N	BSY12048	BS	37°27′37.96″N
		104°47′40.92″E			105°00′0.15″E			104°59′58.32″E
		1619 mH			1329 mH			1329 mH
LHW12015	LC	37°27′4.05″N	BSY12032	BS	37°27′38.54″N	BSY12049	BS	37°27′37.90″N
		104°47′41.63″E			105°00′0.61″E			104°59′58.03″E
		1619 mH			1329 mH			1329 mH
LHW12016	LC	37°27′3.24″N	MSY12033	MS	37°27′38.38″N	CSY12050	CC	37°27′37.99″N
		104°47′41.36″E			105°00′0.81″E			104°59′59.11″E
		1619 mH			1329 mH			1329 mH
MSY12017	MS	37°27′38.52″N	BSY12034	BS	37°27′38.17″N			
		104°59′59.89″E			105°00′0.06″E			
		1329 mH			1329 mH			

*CC, Cyanobacteria-dominated soil crusts; MC, Moss-dominated soil crusts; LC, Lichen-dominated soil crusts; BS, Bare sand*.

### Actinobacteria isolation and maintenance

The following four types of media were prepared to isolate the actionbacterial strains. The main components of the media were as follows: M1 (g l^−1^): glucose 10, yeast extract 1, beef extract 1, casein (enzymatic hydrolysate) 2, agar 15; M2 (g l^−1^): 1/5 strength R2A (Difco); M3 (g l^−1^): cellobiose 2, yeast extract 5, CaCO_3_2, K_2_HPO_4_ 1, MgSO_4_·7H_2_O 0.5, agar 15; M4 (g l^−1^): sodium propionate 2, NH_4_NO_3_ 0.1, KCl 0.1, MgSO_4_·7H_2_O 0.05, agar 15. The isolation media were adjusted to pH 7.2–7.5 using 1 M NaOH and/or 1 M HCl. In addition, betaine (0.125% w/v), sodium pyruvate (0.125% w/v), compound trace salts solution (0.1% v/v), and compound vitamins (0.1% w/v) were added to the media to facilitate the isolation of strains that are difficult to culture (Yue et al., 2006). Aztreonam (25 mg l^−1^) and potassium dichromate (50 mg l^−1^) were also added to the media to prevent or stymie the growth of Gram-stain negative bacteria and fungi that may be present.

The procedure for actinobacteria isolation was carried out as described in Zhang et al. (2010). Briefly, 0.3 ml of 10^−3^ soil suspension was spread on each isolation plate and the plates were incubated at 28°C for 3 weeks. Single colonies were transferred to freshly prepared PYG plates [(g l^−1^) (peptone 3, yeast extract 5, glycerol 10, glycine betaine 1.25, sodium pyruvate 1.25, agar 15, pH 7.5), supplemented with compound trace salts solution (FeSO_4_·7 H_2_O 0.2 g, MnCl·2 H_2_O 0.1 g, ZnSO_4_·7 H_2_O 0.1 g, 0.1% v/v) and compound vitamins (vitamin B1 1 mg, vitamin B2 1 mg, vitamin B3 1 mg, vitamin B6 1 mg, phenylalanine 1 mg, biotin 1 mg, alanine 0.3 mg, 0.1% w/v)] and subsequently purified. The pure cultures were maintained on PYG slants at 4°C and also as glycerol suspensions (20%, v/v) at −80°C.

### Identification of *Geodermatophilaceae*

Purified isolates were transferred to PYG medium and International Streptomyces Project (ISP) medium 2 for observation of the morphological characteristics. Extraction of genomic DNA and PCR amplification of the 16S rRNA gene were performed as described in the methods section of Xu et al. (2003). The purified PCR products were sequenced with an ABI PRISM automatic sequencer. The sequences obtained were compared with available 16S rRNA gene sequences from GenBank using the EzTaxon-e server (http://eztaxon-e.ezbiocloud.net; Kim et al., 2012). The server was used to determine an approximate phylogenetic affiliation of each strain. Multiple alignments with sequences of the related strains and calculations of levels of sequence similarities were carried out using MEGA version 5 (Tamura et al., 2011). A phylogenetic tree was constructed using the neighbor-joining method described in Saitou and Nei (1987). The topology of the phylogenetic tree was evaluated by the bootstrap resampling method of Felsenstein (1985) with 1000 replicates.

### Bioactivity screening

Antimicrobial activities of the isolated strains were investigated by using media containing *Enterococcus faecalis* HH22, *Klebsiella pneumonia* ATCC 700603, *Mycobacterium smegmatis* CPCC240556, *Sporobolomyces salmonicolor* SS04, and *Xanthomonas campestris* pv. *oryzae* PXO99A, respectively, all at a concentration of 10^8^ colony forming units (CFU) per ml. The anti-viral activity against the human immunodeficiency virus (HIV) was investigated using the procedure described in Yang et al. (2013). Results were considered positive if the HIV inhibition ratio was above 90% and at least 80% of the cells survived. This assay was performed under conditions where the sample concentration was 1% (v/v).

### Physiological characteristics determination

From the 70 newly-isolated *Geodermatophilaceae* members, the physiological characteristics were determined for 34 representative strains. Carbohydrate utilization tests were carried out using API 50 CH test kits (bioMérieux) and Biolog GEN III MicroPlates (Biolog Inc.) according to the manufacturer's instructions. Enzymatic activities were determined using API ZYM test kits (bioMérieux) according to the manufacturer's instructions. Bacterial growth was tested at 4, 10, 20, 28, 30, 32, 37, 40, and 45°C on PYG agar medium incubated for 15–30 days. The ability of the strains to grow at different concentrations of NaCl was tested at the following concentrations: 0, 1, 3, and 5–20%, w/v, with 5–20% being tested at intervals of 1.0%. Growth ability in this experiment was determined according to the protocol described by Wang et al. (2001). The pH tolerance was assayed in PYG medium at pH values from 5.0 to 11.0 at intervals of 0.5 pH units. Other physiological and biochemical tests were performed according to the methods established by Williams et al. (1983) and Kämpfer et al. (1991).

The sensitivity of the bacteria to 33 different antibiotics was tested on PYG agar using the following concentrations: amikacin (1500 μg/ml), ampicillin (510 μg/ml), aztreonam (1500 μg/ml), cephalothin (1500 μg/ml), cefazolin (1500 μg/ml), cefepime (1500 μg/ml), cefoperazone (3700 μg/ml), cefotaxime (1500 μg/ml), ceftazidime (1500 μg/ml), ceftriaxone (1500 μg/ml), cefuroxime (1500 μg/ml), chloromycetin (1500 μg/ml), ciprofloxacin (250 μg/ml), clarithromycin (750 μg/ml), clindamycin (100 μg/ml), erythromycin (765 μg/ml), gentamycin (515 μg/ml), gentamycin (6000 μg/ml), levofloxacin (250 μg/ml), macrodantin (15,000 μg/ml), minocycline (1500 μg/ml), norfloxacin (500 μg/ml), ofloxacin (250 μg/ml), oxacillin (50 μg/ml), penicillin G (500 μg/ml), piperacillin (5000 μg/ml), streptomycin (540 μg/ml), streptomycin (15,000 μg/ml), sulfamethoxazole/trimethoprim (1187.5 μg/ml and 62.5 μg/ml), sulfanilamide (15,000 μg/ml), tetracycline (1500 μg/ml), tobramycin (500 μg/ml), and vancomycin (1500 μg/ml).

Numerical comparative analysis of the physiological and biochemical characteristics tested was performed using the NTSYSpc package (version 2.2 for Windows; Exeter Software) (Rohlf, 2000). A binary 0/1 matrix was created based on the positive or negative respective values of 173 physiological characteristics, some of which are described above.

## Results

### Isolation of actinobacteria

A total of 470 purified isolates were obtained in the present study. The 16S rRNA gene sequences revealed that 376 actinobacterial strains were isolated from the 50 samples. These isolates belonged to 18 families and 29 genera, among which the members of *Geodermatophilaceae* were predominant, including 70 strains of three genera. (Supplementary Figure S1). Among the four types of isolation media, M2 resulted in the most successful isolation of actinobacterial strains. Specifically, 35% of the actinobacterial strains were obtained from M2. While 29, 26, and 10% of the actinobacterial isolates were purified from M1, M4, and M3, respectively (Supplementary Figure S2).

The actinobacterial strains, measured in number of isolates per sample, accounted for 35, 30, 24, and 11%, from cyanobacteria-dominated soil crusts, lichen-dominated soil crusts, moss-dominated soil crusts, and bare sands respectively. At the genus level, the diversity of the isolates from the lichen-dominated soil crusts was higher (33%) than cyanobacteria-dominated soil crusts (30.8%) moss-dominated soil crusts (23.6%), and bare sands (12.6%).

### Diversity of *Geodermatophilaceae*

**Figure 1:**
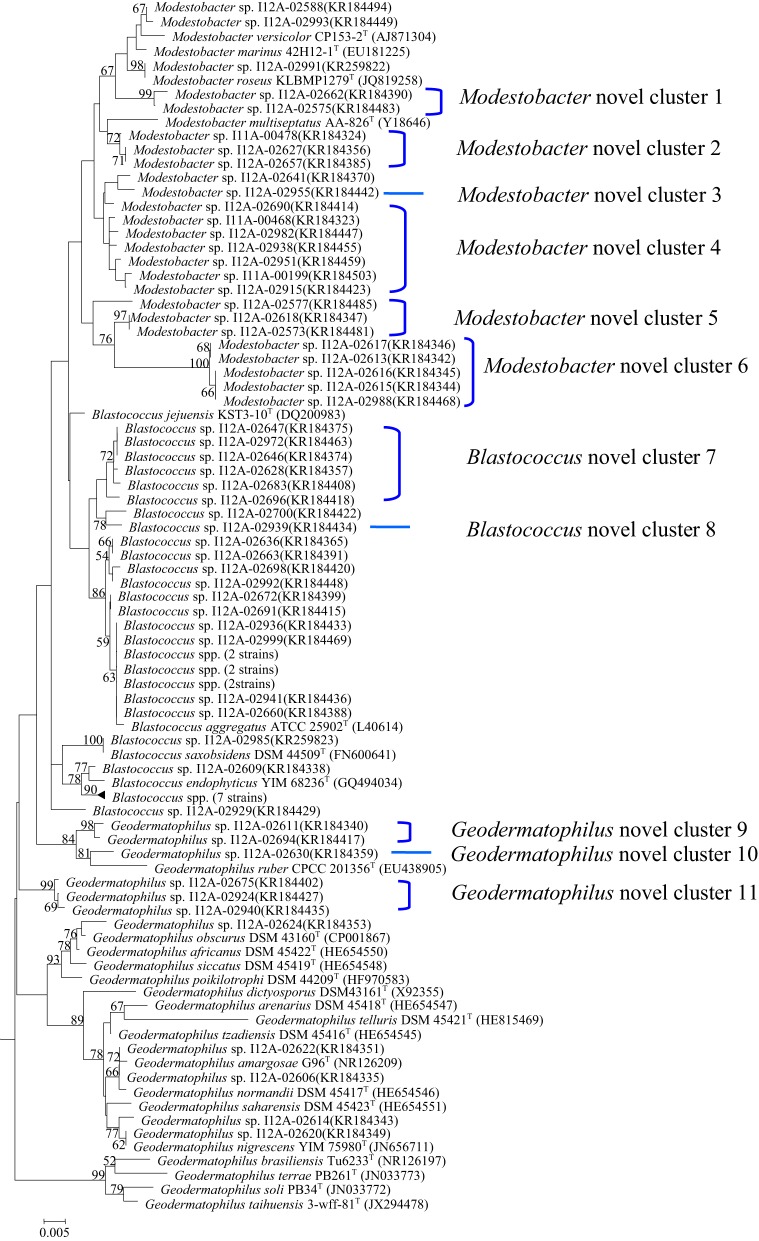
**Phylogenetic dendrogram based on the 16S rRNA gene sequences of the newly-isolated strains and 25 type strains of ***Geodermatophilaceae*****. The sequence of *Kineosporia aurantiaca* 14067^T^ was used as the outgroup. Numbers on branch nodes are bootstrap values. Bar, 1.0% sequence divergence.

In total, 70 *Geodermatophilaceae* strains, including 34 *Blastococcus* spp., 11 *Geodermatophilus* spp., and 25 *Modestobacter* spp. were collected from the 50 samples (Table 2). In the phylogenetic dendrogram based on 16S rRNA gene sequences analysis of the isolates and the type stains of 25 validly described species in the family *Geodermatophilaceae*, these 70 newly-isolated members of the family *Geodermatophilaceae* fell into 23 “species clusters,” with the 16S rRNA gene sequence similarity below 98.65% to the closest homolog as the threshold for differentiating two species (Kim et al., 2014) (Figure 1). As indicated in the phylogenetic dendrogram, six *Modestobacter* “species clusters,” two *Blastococcus* “species clusters” and three *Geodermatophilus* “species clusters” may represent hitherto unrecognized species.

**Table 2:** **Newly-isolated ***Geodermatophilaceae*** members**.

**Strain number**	**Accession number**	**The closest homolog**	**16S rRNA gene similarity with the closest homolog (%)**	**Sample number**	**Sample type**	**Geographical location**	**Isolationmedium**
I12A-02628	KR184357	*Blastococcus aggregatus* ATCC 25902(T)	98.1	MSY12029	MC	G4	M1
I12A-02647	KR184375	*Blastococcus aggregatus* ATCC 25902(T)	98.3	MYW12010	MC	G2	M1
I12A-02683	KR184408	*Blastococcus aggregatus* ATCC 25902(T)	98.4	CSY12047	CC	G4	M3
I12A-02696	KR184418	*Blastococcus aggregatus* ATCC 25902(T)	98.5	CSY12044	CC	G4	M2
I12A-02698	KR184420	*Blastococcus aggregatus* ATCC 25902(T)	99.4	CSY12040	CC	G4	M3
I12A-02663	KR184391	*Blastococcus aggregatus* ATCC 25902(T)	99.4	MSY12019	MC	G4	M3
I12A-02636	KR184365	*Blastococcus aggregatus* ATCC 25902(T)	99.4	MSY12028	MC	G4	M1
I12A-02691	KR184415	*Blastococcus aggregatus* ATCC 25902(T)	99.5	CSY12030	CC	G4	M3
I12A-02992	KR184448	*Blastococcus aggregatus* ATCC 25902(T)	99.5	CSY12040	CC	G4	M1
I12A-02653	KR184381	*Blastococcus aggregatus* ATCC 25902(T)	99.7	BYW12011	BS	G2	M2
I12A-02672	KR184399	*Blastococcus aggregatus* ATCC 25902(T)	99.7	CSY12038	CC	G4	M3
I12A-02689	KR184413	*Blastococcus aggregatus* ATCC 25902(T)	99.7	CSY12040	CC	G4	M3
I12A-02692	KR184416	*Blastococcus aggregatus* ATCC 25902(T)	99.7	CCL12004	CC	G1	M2
I12A-02936	KR184433	*Blastococcus aggregatus* ATCC 25902(T)	99.7	CSY12040	CC	G4	M2
I12A-02941	KR184436	*Blastococcus aggregatus* ATCC 25902(T)	99.7	CSY12040	CC	G4	M1
I12A-02999	KR184469	*Blastococcus aggregatus* ATCC 25902(T)	99.7	CSY12040	CC	G4	M2
I12A-02654	KR184382	*Blastococcus aggregatus* ATCC 25902(T)	99.7	LHW12015	LC	G3	M2
I12A-02660	KR184388	*Blastococcus aggregatus* ATCC 25902(T)	99.7	MHW12014	MC	G3	M2
I12A-02639	KR184368	*Blastococcus aggregatus* ATCC 25902(T)	99.7	MSY12025	MC	G4	M3
I12A-02626	KR184355	*Blastococcus aggregatus* ATCC 25902(T)	99.7	MSY12029	MC	G4	M3
I12A-02666	KR184394	*Blastococcus endophyticus* YIM 68236(T)	98.9	BHW12013	BS	G3	M1
I12A-02971	KR184446	*Blastococcus endophyticus* YIM 68236(T)	98.9	MCL12005	MC	G1	M3
I12A-02953	KR184441	*Blastococcus endophyticus* YIM 68236(T)	99	CSY12027	CC	G4	M2
I12A-02649	KR184377	*Blastococcus endophyticus* YIM 68236(T)	99.1	BHW12012	BS	G3	M2
I12A-02599	KR184501	*Blastococcus endophyticus* YIM 68236(T)	99.1	MSY12019	MC	G4	M1
I11A-00338	KR184318	*Blastococcus endophyticus* YIM 68236(T)	99.1	MSY12025	MC	G4	M2
I12A-02986	KR184467	*Blastococcus endophyticus* YIM 68236(T)	99.2	BSY12034	BS	G4	M1
I12A-02609	KR184338	*Blastococcus endophyticus* YIM 68236(T)	99.6	LHW12015	LC	G3	M2
I12A-02939	KR184434	*Blastococcus jejuensis* KST3-10(T)	98.6	CSY12040	CC	G4	M2
I12A-02700	KR184422	*Blastococcus jejuensis* KST3-10(T)	98.8	CSY12040	CC	G4	M1
I12A-02972	KR184463	*Blastococcus jejuensis* KST3-10(T)	98.8	CSY12040	CC	G4	M2
I12A-02929	KR184429	*Blastococcus jejuensis* KST3-10(T)	98.8	MSY12029	MC	G4	M2
I12A-02646	KR184374	*Blastococcus jejuensis* KST3-10(T)	98.8	MYW12010	MC	G2	M1
I12A-02985	KR259823	*Blastococcus saxobsidens* BC448(T)	99.7	CSY12040	CC	G4	M3
I12A-02622	KR184351	*Geodermatophilus amargosae* G12(T)	99.8	CSY12050	CC	G4	M1
I12A-02606	KR184335	*Geodermatophilus normandii* CF5/3(T)	99.8	MYW12010	MC	G2	M3
I12A-02614	KR184343	*Geodermatophilus nigrescens* YIM 75980(T)	99.5	CSY12030	CC	G4	M4
I12A-02620	KR184349	*Geodermatophilus nigrescens* YIM 75980(T)	100	CSY12044	CC	G4	M2
I12A-02675	KR184402	*Geodermatophilus obscurus* DSM 43160(T)	97.8	CSY12039	CC	G4	M2
I12A-02940	KR184435	*Geodermatophilus obscurus* DSM 43160(T)	97.8	CSY12040	CC	G4	M1
I12A-02924	KR184427	*Geodermatophilus obscurus* DSM 43160(T)	98	CCL12004	CC	G1	M2
I12A-02624	KR184353	*Geodermatophilus obscurus* DSM 43160(T)	99.1	MSY12029	MC	G4	M3
I12A-02694	KR184417	*Geodermatophilus ruber* CPCC 201356(T)	97.8	CSY12030	CC	G4	M3
I12A-02611	KR184340	*Geodermatophilus ruber* CPCC 201356(T)	97.8	LHW12016	LC	G3	M3
I12A-02630	KR184359	*Geodermatophilus ruber* CPCC 201356(T)	98.4	CSY12050	CC	G4	M1
I12A-02982	KR184447	*Modestobacter marinus* 42H12-1(T)	97.8	CSY12040	CC	G4	M1
I11A-00468	KR184323	*Modestobacter marinus* 42H12-1(T)	97.8	CSY12040	CC	G4	M1
I12A-02690	KR184414	*Modestobacter marinus* 42H12-1(T)	98.1	CSY12030	CC	G4	M1
I12A-02938	KR184455	*Modestobacter marinus* 42H12-1(T)	98.1	CSY12040	CC	G4	M1
I12A-02915	KR184423	*Modestobacter marinus* 42H12-1(T)	98.1	MSY12017	MC	G4	M2
I12A-02627	KR184356	*Modestobacter marinus* 42H12-1(T)	98.2	CSY12027	CC	G4	M1
I12A-02662	KR184390	*Modestobacter marinus* 42H12-1(T)	98.2	MCL12002	MC	G1	M3
I12A-02951	KR184459	*Modestobacter marinus* 42H12-1(T)	98.3	CSY12040	CC	G4	M1
I11A-00199	KR184503	*Modestobacter marinus* 42H12-1(T)	98.3	CSY12050	CC	G4	M2
I12A-02657	KR184385	*Modestobacter marinus* 42H12-1(T)	98.6	MHW12014	MC	G3	M1
I12A-02575	KR184483	*Modestobacter marinus* 42H12-1(T)	98.6	MSY12029	MC	G4	M4
I12A-02993	KR184449	*Modestobacter marinus* 42H12-1(T)	99.4	LYW12008	LC	G2	M2
I12A-02588	KR184494	*Modestobacter marinus* 42H12-1(T)	99.5	BSY12032	BS	G4	M4
I12A-02613	KR184342	*Modestobacter multiseptatus* AA-826(T)	96.1	CSY12040	CC	G4	M2
I12A-02616	KR184345	*Modestobacter multiseptatus* AA-826(T)	96.3	CSY12040	CC	G4	M2
I12A-02615	KR184344	*Modestobacter multiseptatus* AA-826(T)	97	CSY12040	CC	G4	M1
I12A-02617	KR184346	*Modestobacter multiseptatus* AA-826(T)	97.1	CSY12040	CC	G4	M2
I12A-02988	KR184468	*Modestobacter multiseptatus* AA-826(T)	97.2	CCL12004	CC	G1	M2
I12A-02577	KR184485	*Modestobacter multiseptatus* AA-826(T)	97.5	CSY12027	CC	G4	M1
I12A-02573	KR184481	*Modestobacter multiseptatus* AA-826(T)	97.8	BSY12026	BS	G4	M2
I12A-02618	KR184347	*Modestobacter multiseptatus* AA-826(T)	97.8	MSY12029	MC	G4	M2
I11A-00478	KR184324	*Modestobacter multiseptatus* AA-826(T)	98.8	MSY12029	MC	G4	M1
I12A-02991	KR259822	*Modestobacter roseus* KLBMP 1279(T)	100	MSY12025	MC	G4	M4
I12A-02955	KR184442	*Modestobacter versicolor* CP153-2(T)	98.6	MHW12014	MC	G3	M1
I12A-02641	KR184370	*Modestobacter versicolor* CP153-2(T)	98.8	BYW12009	BS	G2	M4

*CC, Cyanobacteria-dominated soil crusts; MC, Moss-dominated soil crusts; LC, Lichen-dominated soil crusts; BS, Bare sand. G1, 37°25′37″−37°25′40″N, 104°35′7″−104°35′10″ E, ~1700 mH; G2, 37°25′29″−37°25′32″N, 104°43′51″−104°43′54″ E, ~1700 mH; G3, 37°27′3″−37°27′5″N, 104°47′40″−104°47′43″ E, ~1620 mH; G4, 37°27′37″−37°27′40″N, 104°59′58″−105°0′1″ E, ~1330 mH*.

### Bioactivities of newly-isolated strains

Among the 70 *Geodermatophilaceae* strains, 3 exhibited activity against Enterococcus faecalis (4.3%), 3 against Klebsiella pneumonia (4.3%), 4 against Mycobacterium smegmatis (5.7%), 6 against Sporobolomyces salmonicolor (8.6%), 2 against Xanthomonas campestris pv. oryzae PXO99A (2.9%), and 6 against HIV (8.6%), respectively. Additionally, 9 of the isolates exhibited activities in more than one of these screening models. In total, 19.7% of the newly-isolated *Geodermatophilaceae* strains showed activity in at least one of the six antibiotic screens.

### Physiological characteristics of newly-isolated strains

**Figure 2:**
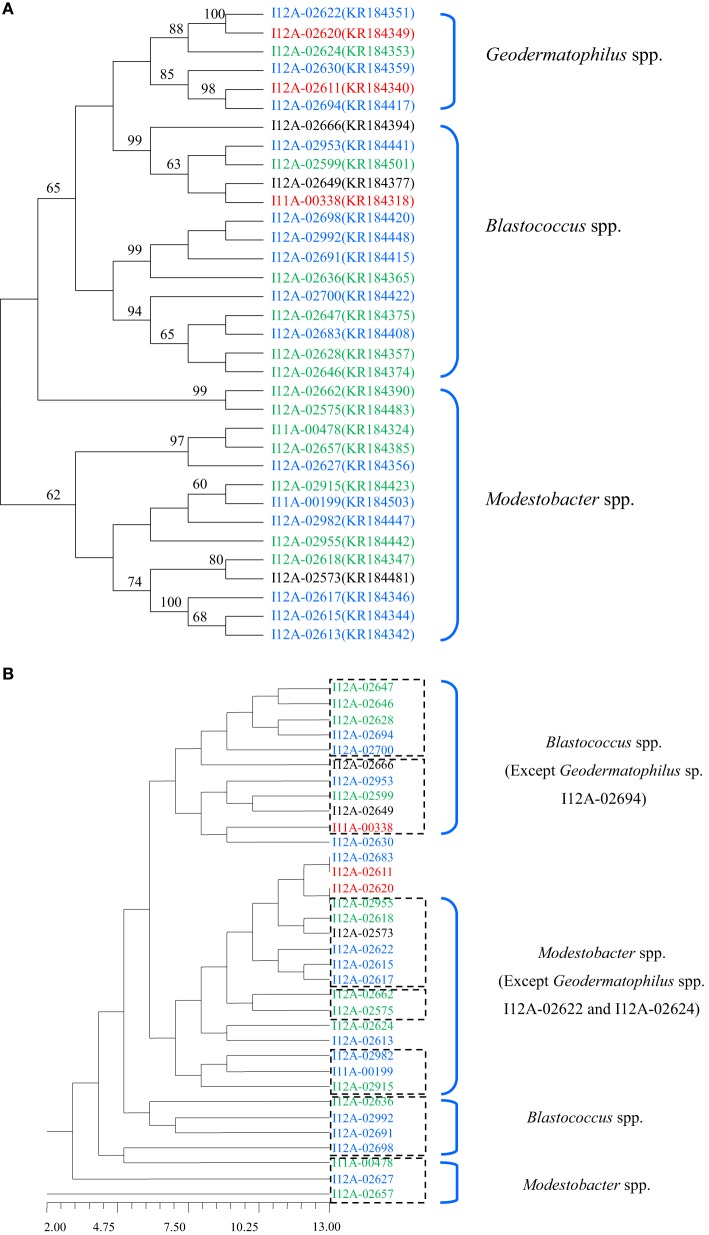
**(A)** Dendrogram based 16S rRNA gene sequences analysis of the tested strains. **(B)** Dendrogram based on the physiological characteristics profiles of the tested strains. Different colors denote the strains isolated from different types of samples. Blue, Cyanobacteria-dominated soil crusts; Green, Moss-dominated soil crusts; Red, Lichen-dominated soil crusts; Black, Bare sand.

The strains assayed for physiological characteristics were similar in their physiological characteristic profiles in the following capacity: more than 60% of the strains tested could utilize dextrin, D-fructose, D-fructose-6-PO_4_, D-galactose, α-D-glucose, glucuronamide, α-keto-glutaric acid, D-malic acid, D-maltose, D-mannose, D-trehalose, D-turanose and sucrose as their sole carbon source, and 91% of the strains tested assimilated esculin ferric citrate and potassium 5-ketogluconate and produced acid. In the API ZYM assay, none of the strains tested was positive for β-fucosidase, N-acetyl-β-glucosaminidase, or α-mannosidase. Twenty-nine strains showed the enzymatic activities of acid phosphatase, alkaline phosphatase, esterase (C4), esterase lipase (C8), leucine arylamidase, lipase (C14), and valine arylamidase. Most of the tested strains were resistant to aztreonam (1500 μg/ml), sulfanilamide (15,000 μg/ml), and sulfamethoxazole/trimethoprim (1187.5 μg/ml and 62.5 μg/ml). The phylogenetic dendrogram based on 173 physiological characteristics of the tested strains showed that the micro-ecological environment from which the strains were isolated was an important factor correlating with the physiological characteristic profiles of the isolates. The strains exhibited characteristics specific to the micro-ecological environment where they were found (Figure 2).

## Discussion

The family *Geodermatophilaceae* is a newly-established actinobacterial taxon. Normand et al. (1996) proposed the family *Geodermatophilaceae* in 1996, which was regarded as an invalid taxon at that time. In 2006, based on the common characteristics of the genera *Geodermatophilus, Blastococcus*, and *Modestobacter*, Normand (2006) summarized the typical characteristics of *Geodermatophilaceae*. Subsequently, the family *Geodermatophilaceae* was finally accommodated as a validly described taxon in the phylum *Actinobacteria*. To date, the family *Geodermatophilaceae* consists of three genera: *Geodermatophilus, Blastococcus*, and *Modestobacter*, that includes 25 validly described species.

The members of *Geodermatophilaceae* were found from various environments, including soil samples (Zhang et al., 2011; Jin et al., 2013), soil crusts (Reddy et al., 2007), deep sub-seafloor sediment (Ahrens and Moll, 1970), even stone habitats (Salazar et al., 2006; Chouaia et al., 2012; Gtari et al., 2012; Normand et al., 2012), dry-hot valley (Nie et al., 2012), and arid sand from desert (Montero-Calasanz et al., 2012, 2013a,b,c). In this study, we found *Geodermatophilaceae* members ubiquitously in desert soil samples, and we obtained *Geodermatophilaceae* cultures from three different types of desert soil crusts, as well as from the bare sands. These four environments represent typical micro-ecological environments in the Shapotou region. As we have observed, most *Geodermatophilaceae* members could form tiny motile spores or dormant spores, allowing them to spread around and survive long periods of desiccation. Moreover, most of the *Geodermatophilaceae* members we tested formed pink to black colonies on different types of agar plates. The pigmentation, cell wall composition and a high G+C content may increase protection of these strains from UV damage in the desert environments, where the UV transparency is often high.

The abundance and ubiquitous distribution of the *Geodermatophilaceae* in desert environments exhibited in relation to their resident microbiota, and in turn, the micro-ecological environments endowed the microorganisms with some specific metabolic characteristics. We found that the abundance and diversity of the *Geodermatophilaceae* in lichen- and cyanobacteria-dominated soil crusts were much higher than those of the bacteria found in moss-dominated soil crusts or bare sands. In the desert, the moisture, organic, and nitrogen content of the soil were the vital factors in determining physiological characteristics of the organisms. The lichen- and cyanobacteria-dominated soil crusts may contain a much higher proportion of clay and humic colloidal material, which can markedly affect the physiological activities of the strains from different micro-ecological environments.

The assayed physiological characteristics of the *Geodermatophilaceae* also showed a probable relationship with the resident microbes of the respective micro-ecological environments. In the dendrogram based on 173 physiological characteristics of the 34 tested *Geodermatophilaceae* strains, strains from the same micro-ecological environment were more likely to gather closely. The clusters shown in the phylogenetic dendrogram based on 16S rRNA gene sequences were interrupted in the dendrogram based on the physiological characteristics profile, which indicated that the micro-ecological environments where the strains were isolated significantly influenced the physiological characteristic profiles of the isolates (Figure 2).

Compared to our previous study and other related studies in the literature, we discovered many interesting diverse bioactivities for rare actinobactieria, which may be caused by characteristics of the extreme environments where these strains were found. Isolation and analysis of the bioactive compounds underlying these bioactivities will provide more detailed information on the mechanism of these activities. In this context, the members of the family *Geodermatophilaceae* are found to be the biological pioneers in extreme environments, especially in extreme arid environments. Further study on the cultures in this family will be advantageous to those seeking to understand mechanisms of environmental stress resistance, desertification control, and environmental remediation. In addition, studying these organisms will aid in the discovery of novel metabolic compounds.

### Conflict of interest statement

The authors declare that the research was conducted in the absence of any commercial or financial relationships that could be construed as a potential conflict of interest.

## Supplementary material

Supplementary Figure S1 **Phylogenetic dendrogram based on 16S rRNA gene sequences analysis of the isolates**. Bootstrap values >50% (based on 1000 resampled datasets) are shown at branch nodes. Bar, 0.02 substitutions per site.

Supplementary Figure S2 **The number of actinobacteria in the different samples**. Sample numbers were ordered from left to right (x-axis) according to the sample number in Table 1, and numbers of actinobacterial colonies from bottom to top (y-axis) were estimated and ordered according to the colony numbers that appeared on isolation medium of M4, M3, M2, and M1.
