# Supplementary material for: Ubiquity, diversity and physiological characteristics of Geodermatophilaceae in Shapotou National Desert Ecological Reserve
Source: Front Microbiol. 2015 Sep 30;6:1059. doi: 10.3389/fmicb.2015.01059 (PMC4588033; doi:10.3389/fmicb.2015.01059)

**Supplementary Fig S1.**

*Pseudonocardia* spp.

*Kibdelosporangium* spp.

*Actinophytocola* spp.

*Actinomycetospora* spp.

*Rhodococcus* sp. I12A-02688

*Mycobacterium* spp.

*Saccharothrix* spp.

***Modestobacter* spp.**

***Blastococcus* spp.**

***Geodermatophilus* spp.**

*Cryptosporangium* spp.

*Kineosporia* spp.

*Micrococcus* sp. I12A-02604

*Arthrobacter* sp. I12A-02637

*Phycicoccus* sp. I12A-02932

*Okibacterium* spp.

*Promicromonospora* sp. I12A-02568

*Cellulomonas* spp.

*Actinoplanes* sp. I12A-02925

*Micromonospora* sp. I12A-02540

*Micromonospora* sp. I12A-02944

*Micromonospora* spp.

*Asanoa* sp. I12A-02658

*Micromonospora* spp.

*Actinopolymorpha* sp. I12A-02601

*Nocardioides* sp. I12A-02619

*Kribbella* spp.

*Streptosporangium* sp. I11A-00471

*Streptomyces* spp.

*Nonomuraea* spp.

*Actinomadura* sp. I12A-02643

*Glycomyces* spp.

100

100

100

100

100

95

100

100

89

98

94

73

67

74

99

50

77

64

97

100

79

83

95

63

64

94

99

64

88

68

65

62

0.02

**Supplementary Fig S2.**


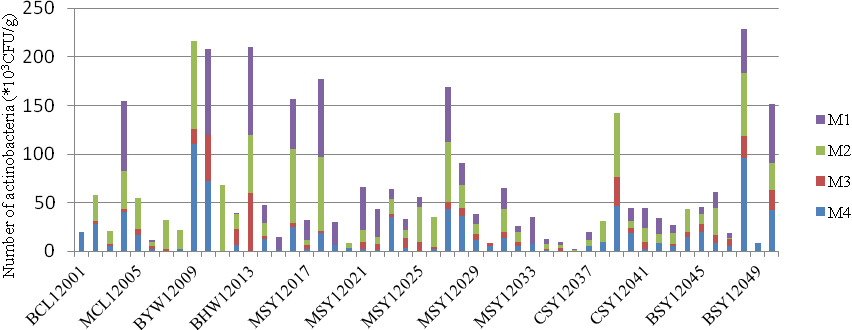

Supplement: Supplementary Figure S1 — Phylogenetic dendrogram based on 16S rRNA gene sequences analysis of the isolates. Bootstrap values >50% (based on 1000 resampled datasets) are shown at branch nodes. Bar, 0.02 substitutions per site. [file DataSheet1.DOC]
